# Supplementary material for: Analysis of Exertion-Related Injuries and Fatalities in Laborers in the United States
Source: Int J Environ Res Public Health. 2023 Feb 2;20(3):2683. doi: 10.3390/ijerph20032683 (PMC9916328; doi:10.3390/ijerph20032683)
Supplement: Supplementary file 1 [file ijerph-20-02683-s001.zip › ijerph-2114203-supplementary.pdf]

International Journal of
Environmental Research and Public Health

Table S1. Occupational Injury and Illness Classification System Codes.

**Definition of Severe Injury Categories**	
Topics	Codes
Heat-related Injury	Effects of heat and light; Effects of heat and light, unspecified; heat stroke; heat syncope; heat fatigue; heat edemia; heat exhaustion, prostration; Multiple effects of heat and light; Effects of heat and light, n.e.c (172)
Cold-related Injury	Effects of reduced temperature; Effects of reduced temperature, unspecified; Frostbite; Hypothermia; Multiple effects of reduced temperature; Effects of reduced temperature, n.e.c (173)
Anemia	Anemia (21)
Anaphylactic Shock	Anaphylactic shock, anaphylaxsis (1962)
Pulmonary Embolism	Pulmonary Embolism
Cardiac	Myocardial infarction (heart attack, 2331); Ischemic heart disease, including heart attack; Ischemic heart disease, unspecified; Ischemic heart disease, n.e.c; Angina (2332)

Table S2. Example Keywords Used to Classify Exertion-related Injuries.

Heat illness, heat injury, heat stroke, fainting, dehydration, heat exhaustion, heat fatigue, cold injury, frostbite, hypothermia, anaphylactic shock, cardiac event, ischemic heart disease, heart attack, myocardial infarction, pulmonary embolism, heart disease, anaphylaxis, angina, sickle cell, blood clot, asthma, infection, commotio cordis.
